# Evaluation of serial erythrocyte sedimentation rate and C-reactive protein monitoring in infectious disease outpatient parenteral antimicrobial therapy patients

**DOI:** 10.1017/ash.2025.15

**Published:** 2025-03-03

**Authors:** Katarina Jackson, John J. Veillette, Jared Olson, Allan M. Seibert, Brandon J. Webb

**Affiliations:** 1 Department of Pharmacy, Intermountain Medical Center, Murray, UT, USA; 2 Infectious Diseases Telehealth Service, Intermountain Health, Murray, UT, USA; 3 Department of Pharmacy, Primary Children’s Hospital, Salt Lake City, UT, USA; 4 Division of Infectious Diseases, Department of Pediatrics, University of Utah, Salt Lake City, UT, USA; 5 Division of Infectious Diseases and Clinical Epidemiology, Intermountain Health, Salt Lake City, UT, USA

## Abstract

Of 313 patients whose outpatient parenteral antimicrobial therapy was managed by an ID physician, only 39 [12.5%, 95% CI (8.8%–16.1%)] had clinical decisions influenced by erythrocyte sedimentation rate (ESR), C-reactive protein (CRP), or both. ESR/CRP ordering was associated with $530 in excess cost per treatment course (average duration 5.1 weeks) representing a diagnostic stewardship opportunity.

## Introduction

Outpatient parenteral antimicrobial therapy (OPAT) is often used as an effective, less costly alternative to inpatient treatment of severe infections.^1^ To ensure OPAT safety, both the Infectious Disease Society of America (IDSA) and UK Good Practice Recommendations endorse serial monitoring of complete blood cell counts, renal panels, and liver function tests.^1,2^ However, these guidelines lack recommendations for monitoring of inflammatory markers for efficacy [e.g. erythrocyte sedimentation rate (ESR), C-reactive protein (CRP)]. While ESR and CRP assist in the initial diagnosis of several infectious conditions,^3–5^ the utility of serial monitoring is unknown. To our knowledge, repeat ordering of ESR/CRP is only recommended in two IDSA guidelines: prosthetic joint infections (at the end of treatment prior to 2^nd^ stage revision), and vertebral osteomyelitis (after 4 weeks of antimicrobial therapy).^3,4^ IDSA’s diabetic foot infection guideline neither recommends for nor against serial ESR/CRP monitoring, citing only one study that found that CRP failed to predict treatment failures.^5,6^ In our own healthcare system (in the absence of standardized OPAT monitoring guidelines), we have observed the ordering of weekly ESR/CRP for various indications, which might represent a diagnostic stewardship opportunity. Herein, we evaluate the utility of serial ESR/CRP monitoring for clinical decision-making in OPAT management.

## Methods

We identified unique patients (any age) who were discharged on OPAT between 11/2022–4/2023 from one of 23 Intermountain Health (IH) hospitals, followed by an IH infectious diseases (ID) physician and had at least 2 ESRs or 2 CRPs ordered by the same ID physician within 6 weeks of discharge. Patients were then excluded for the following reasons: no ID clinic note after discharge, autoimmune condition (identified by ICD-10 code), antiviral or antifungal OPAT, deceased prior to finishing OPAT, or inadequate information in the ID clinic note to determine clinical management. Hospital course, comorbidities, demographics, laboratory values, and microbiology data were extracted electronically, whereas OPAT indication (selected from a standardized list) and aspects of clinical management were abstracted manually from the electronic medical record (EMR).

The primary outcome was the percentage of patients for whom an ID physician documented in the chart that ESR and/or CRP influenced decision-making, defined as a change in antibiotics (duration, dose, route, or regimen) or any other aspect of clinical management (e.g. ordering repeat imaging or labs, or arranging future visits). Subgroup analyses were performed to assess provider ordering variability and to stratify decision-making by OPAT indication. Lastly, we described the percentage of ESR/CRPs that were within normal limits, and direct lab costs (cost of lab test plus labor) associated with monitoring that did not appear to influence clinical decisions within 2 weeks following the test result (i.e. excess lab orders). Descriptive statistics were utilized in all analyses. This study was exempted by the IH Institutional Review Board as a quality improvement project.

## Results

**Figure 1.**
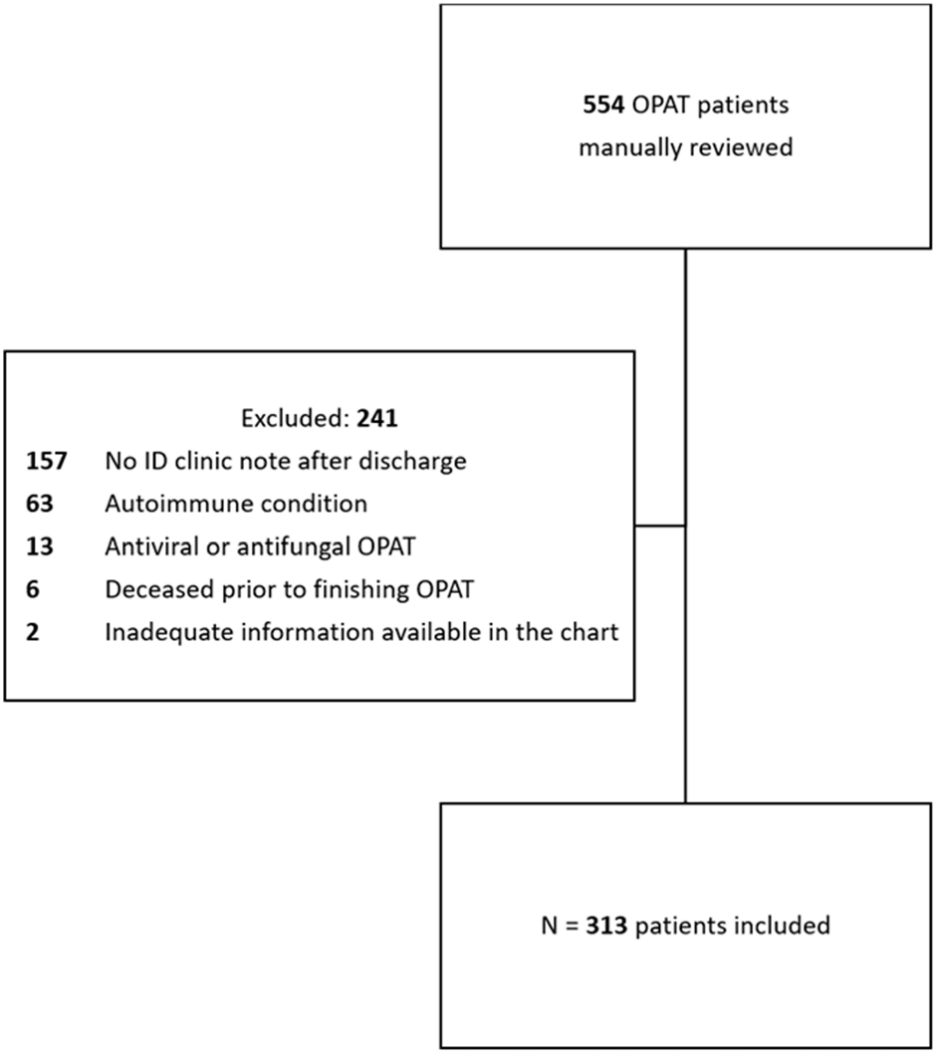
Inclusion/exclusion criteria. Abbreviations: CRP, C-reactive protein; ESR, erythrocyte sedimentation rate; ID, infectious diseases; OPAT, outpatient parenteral antimicrobial therapy.

Of 554 patients reviewed, 313 (56.5%) met inclusion criteria (Figure 1). Most patients were male (196 [62.6%]) with a median age of 62 (IQR 47–72). The most common indications for OPAT were septic arthritis (93 [29.7%]), osteomyelitis (91 [29.1%]), and endocarditis (33 [10.5%]). Demographics, baseline characteristics, lab ordering, clinical decision-making, and ID Clinic visit details are included in Table 1. Based on EMR documentation, 39/313 patients (12.5%, [95% CI 8.8%–16%]) had a clinical decision influenced by ESR, CRP, or both at any time during their OPAT course. Notably, inflammatory markers were most often used in combination with other clinical factors to decide on management: few patients had decisions made based on ESR only (6 [1.9%]) or CRP only (4 [1.3%]). Most clinical decisions based on ESR only (5/6, 83%) or CRP only (3/4, 75%) were in bone and joint infections (osteomyelitis or septic arthritis).

**Table 1.** Demographics, antibiotic treatment, and outcomes

Variable	All patients, N = 313 (%)
Age, median (IQR), years	62 (47–72)
Female sex	117 (37)
Charlson comorbidity score, median (IQR)	5 (2–8)
Treatment duration, median (IQR), weeks	6 (4–6)
**OPAT Indication**	
Septic arthritis	93 (29.7)
Osteomyelitis	91 (29.1)
Endocarditis	33 (10.5)
Other	21 (6.7)
SSTI	17 (55.4)
Genitourinary	16 (5.1)
CNS	14 (4.5)
Intra-abdominal	14 (4.5)
Unknown	9 (2.9)
IV-line infection	5 (1.6)
**Admit Facility**	
Large Hospital (≥ 200 beds)	269 (85.9)
**Primary Outcomes**	
ESR, CRP, or both influenced decision-making	39 (12.5)
Decisions made based on ESR only	6 (1.9)
Decisions made based on CRP only	4 (1.3)
*Description of changes to clinical management*	
Antibiotic duration extended	37 (11.8)
Antibiotics escalated	8 (2.6)
New imaging obtained	1 (0.3)
Antibiotic duration shortened	1 (0.3)
IV to PO conversion	3 (1)
**ESR/CRP Ordering Among ID providers (n = 25)^a^**	
CRP orders per patient	
Range	1–17
Median (IQR)	4.7 (1.7–5.9)
ESR orders per patient	
Range	0–17
Median (IQR)	4.3 (3.8–5.1)
Outpatient ID Clinic Visits for OPAT	
Range	1–3
Median (IQR)	2 (1–2)

CRP, C-Reactive Protein; ESR, Erythrocyte Sedimentation Rate; IQR, interquartile range; IV, intravenous; OPAT, outpatient parenteral antimicrobial therapy; PO, by mouth.

a All 25 ID providers, all OPAT indications.

There was notable variability in inflammatory marker ordering among the 25 included ID providers (Table 1, all OPAT indications). This variability persisted when limiting the evaluation to bone and joint infections only (n = 20 ID providers): CRP – range 1–17 orders per OPAT course, median 5.7 (IQR 4.5–6.1); ESR – range 0–17 orders per OPAT course, median 4.9 (IQR 4.2–5.7).

Of 1,336 ESRs obtained, 434 (32.4%) were within normal limits (≤ 20 mm/hr) and of 1,575 CRPs obtained, 811 (51.5%) were within normal limits (≤ 1 mg/dL). Evaluating specific OPAT indications, 10.5% (52/493), 20.1% (96/478), and 5.5% (5/91) of ESRs versus 10.1% (54/535), 22% (117/533), and 5.6% (8/143) of CRPs contributed to decision-making for septic arthritis, osteomyelitis, and endocarditis, respectively. There was no evidence that ESR/CRP-related decisions were made for any other OPAT indication. Across all infection types, an average of 5 excess CRPs and 4 excess ESRs per OPAT course (average 5.1 weeks) were ordered (estimated average excess cost $530 per patient; $165,890 for 313 patients in 6 months, or $331,780 per year).

## Discussion

Only 12.5% of serial ESR and CRP orders appeared to influence clinical decision-making in our ID OPAT patients. Several factors likely contributed to these findings, and we offer the following suggestions to optimize ESR/CRP ordering based on our data: First, ESR and CRP were ordered weekly, but clinic visits with the ID physician occurred less often (generally 1–2 visits per OPAT course). In conditions where ESR/CRP trending is indicated, clinicians should consider decreasing monitoring frequency to preceding a clinical visit or at the start and end of therapy (although this may represent operational challenges). Second, up to 30% of ESRs and 50% of CRPs were within normal limits. Discontinuing ESR/CRP after normalization is likely low-hanging fruit for diagnostic stewardship. Third, ESR/CRP values did not appear to influence decision-making outside of bone and joint infections or endocarditis, and perhaps these labs should be avoided for other OPAT indications. Lastly, ESR did not appear to add value to CRP when both were ordered. Because ESR takes longer to normalize and CRP is a better measure of acute-phase response,^7^ we intend to remove ESR from our OPAT note template.

While our study uniquely evaluated the utility of serial ESR/CRP ordering in ID OPAT patients, it had many limitations. We relied on EMR documentation to provide the rationale behind clinical decisions, and it is possible that decisions were made without chart documentation. Furthermore, while these data reflect our healthcare system’s practices, it is possible that ID physicians elsewhere rely more on ESR/CRP when making decisions. We did not separate orthopedic hardware-related infections from native bone and joint infections, a distinction that is needed in future studies on this topic. We also did not identify malignancies that might influence ESR/CRP values. We did not evaluate appropriateness of clinical decisions, and it is possible that some decisions (e.g. extending antibiotics based on ESR only) might have been unnecessary; thus, limiting OPAT ESR/CRP surveillance might also have potential as an antibiotic stewardship intervention. Chart review was completed by one independent reviewer, who was not blinded to the study objectives. Finally, we did not correlate lab monitoring or decision-making with clinical outcomes, which is an important area of future study. Such studies are needed so OPAT guidelines can recommend specific frequencies of lab monitoring based on safety and efficacy data.

In summary, ESR and CRP rarely influenced clinical decision-making in our ID OPAT patients and were associated with substantial costs. This likely represents a diagnostic stewardship opportunity to decrease excess monitoring and costs for patients and healthcare systems.
